# Supplementary figures and images for: The Ceramide-Dependent EV Secretome Differentially Affects Prostate Cancer Cell Migration
Source: Cells. 2025 Apr 4;14(7):547. doi: 10.3390/cells14070547 (PMC11988362; doi:10.3390/cells14070547)

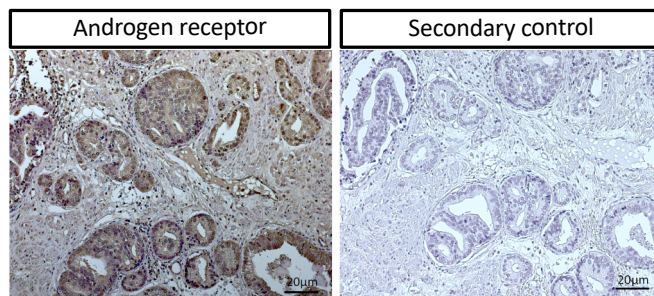

**Supplementary Figure S1**  
**Expression of SMPD2 and SMPD3 in prostate cancer**

Supplement: Supplementary file 1 [file cells-14-00547-s001.zip › Supplementary FigureS1.pdf]

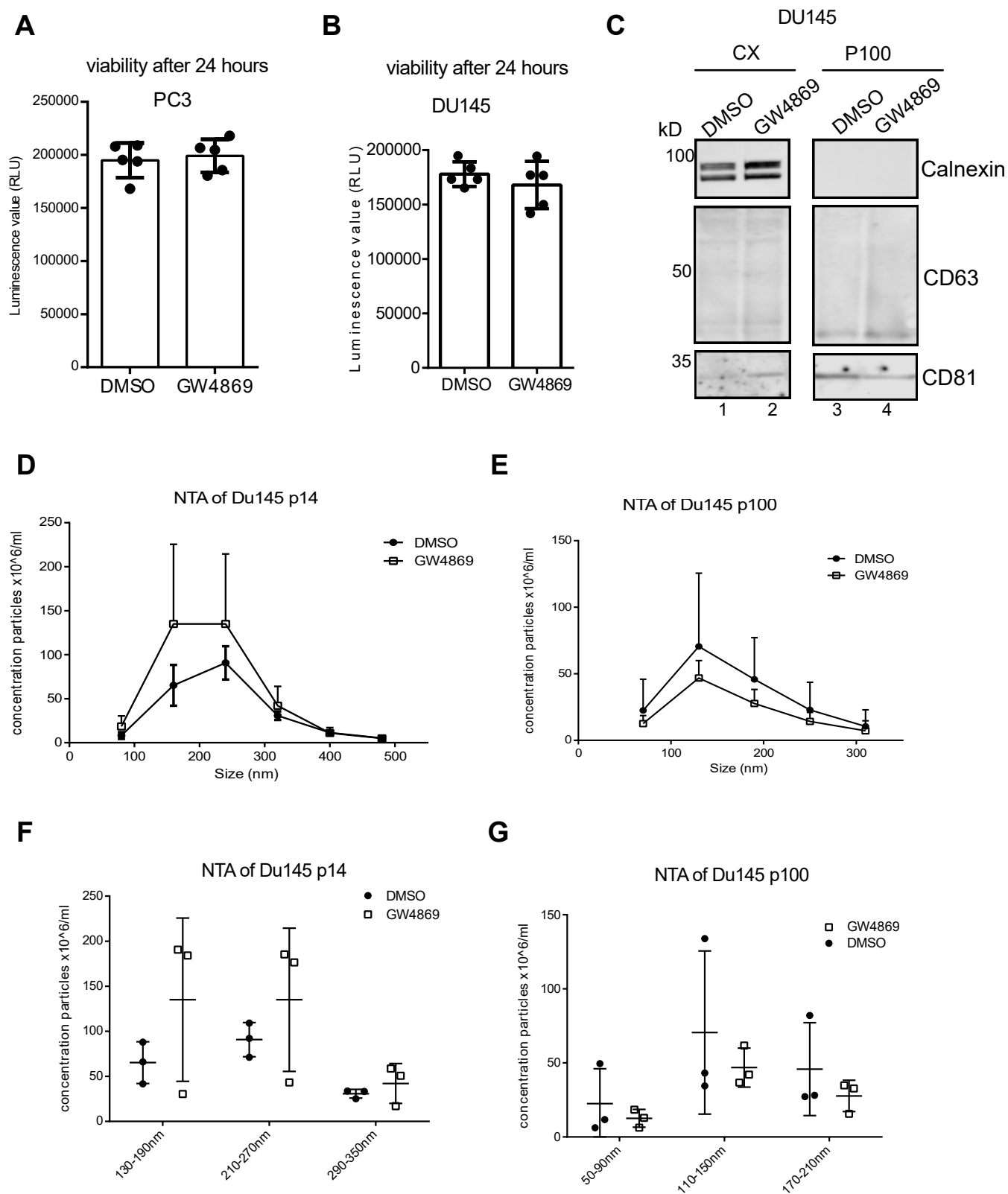

**Supplementary Figure S2**  
**NSMases-dependent EV secretion from DU145 cells**

Supplement: Supplementary file 1 [file cells-14-00547-s001.zip › Supplementary FigureS2.pdf]

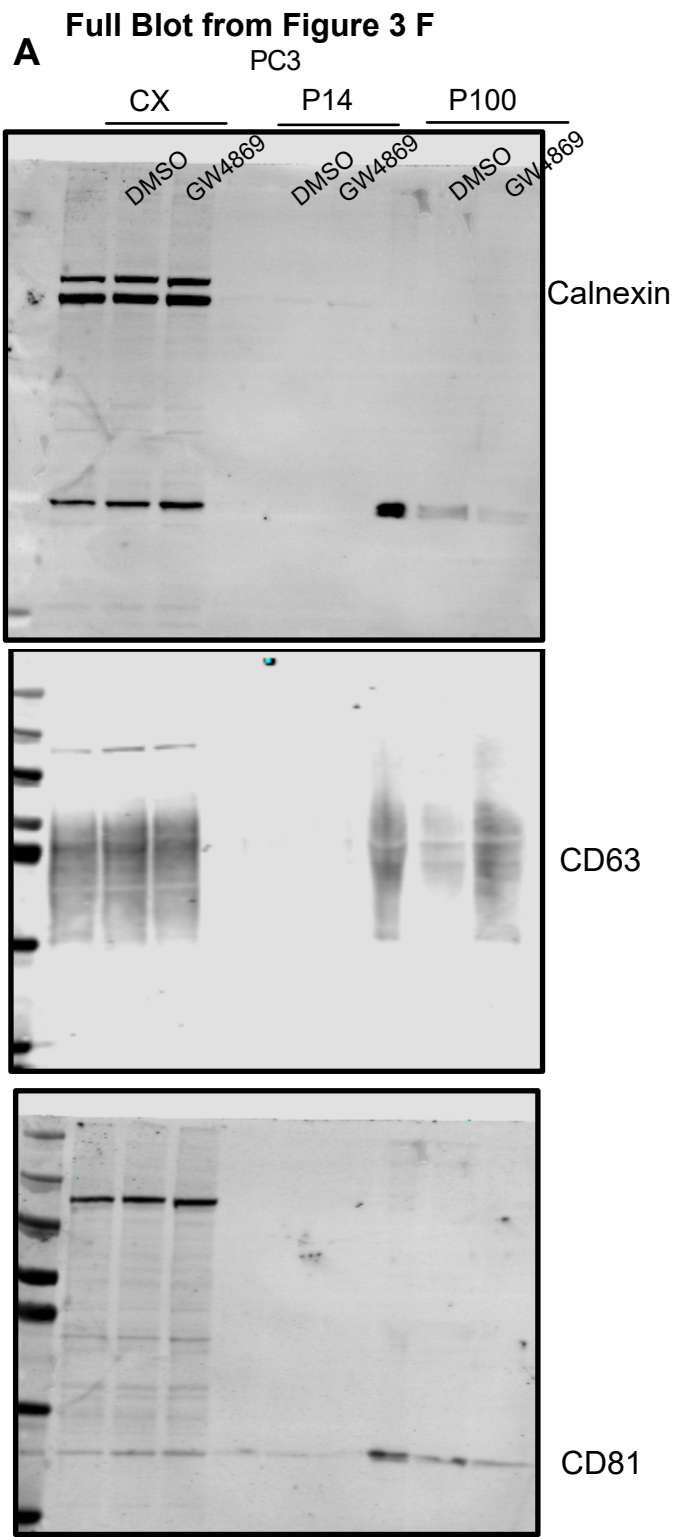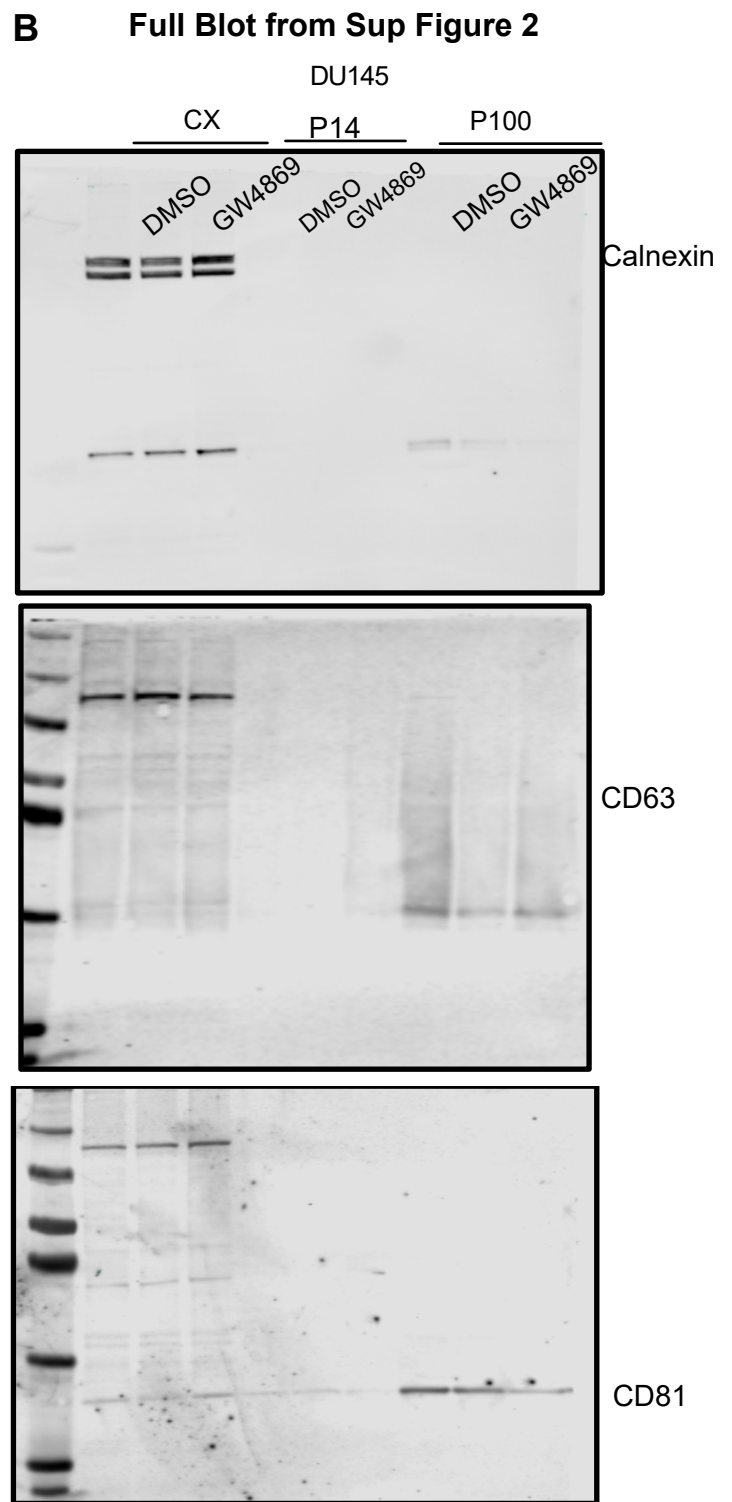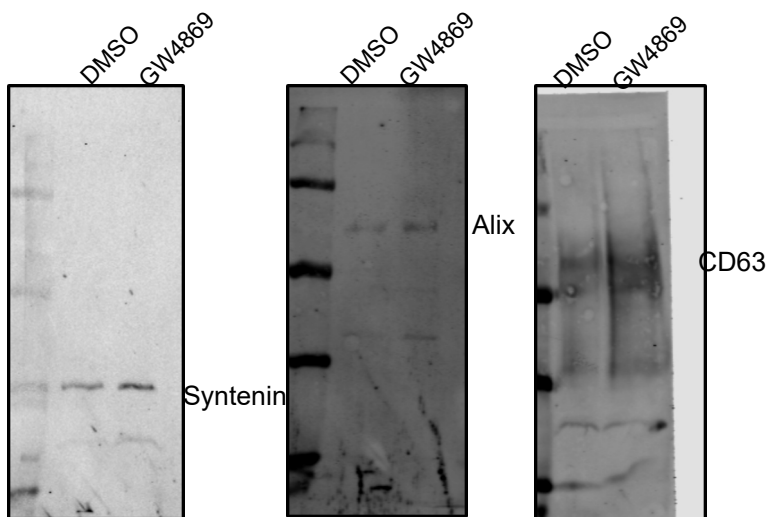

Supplementary Figure S3 Full Western Blots

Supplement: Supplementary file 1 [file cells-14-00547-s001.zip › Supplementary FigureS3.pdf]
